# Design and clinical developments of aptamer-drug conjugates for targeted cancer therapy

**DOI:** 10.1186/s40824-021-00244-4

**Published:** 2021-11-25

**Authors:** Do-Hun Kim, Jin-Myung Seo, Kyung-Ju Shin, Su-Geun Yang

**Affiliations:** 1 Department of Biomedical Science, BK21 FOUR Program in Biomedical Science and Engineering, Inha University College of Medicine, Incheon, 22212 South Korea; 2 Inha Institute of Aerospace Medicine, Inha University College of Medicine, Incheon, 22332 South Korea

**Keywords:** Aptamers, Aptamer drug conjugates (ApDCs), Cancer therapy, Targeted treatment

## Abstract

**Background:**

Aptamer has been called “chemical antibody” which displays the specific affinity to target molecules compared to that of antibodies and possesses several therapeutic advantages over antibodies in terms of size, accessibility to synthesis, and modification. Based on the attractive properties, aptamers have been interested in many directions and now are emerged as new target-designed cancer drug.

**Main body:**

Currently, new types of aptamers have been reported and attracted many scientists’ interesting. Due to simplicity of chemical modification and ready-made molecular engineering, scientists have developed newly designed aptamers conjugated with a wide range of therapeutics, aptamer-drug conjugates; ApDCs, from chemotherapy to phototherapy, gene therapy, and vaccines. ApDCs display synergistic therapeutic effects in cancer treatment.

**Conclusion:**

In this paper, we reviewed various kinds of ApDCs, i.e., ApDC nucleotide analogs, ApDC by drug intercalation, and ApDC by using chemical linker. Current data prove these ApDCs have sufficient potential to complete clinical development soon. Advanced technology of cancer drug delivery and combination treatment of cancers enables aptamer and conjugated drug (ApDCs) efficient means for targeted cancer treatment that reduces potential toxicity and increases therapeutic efficacy.

## Introduction

An aptamer is a tertiary structural nucleic acid capable of binding to a target molecule with high affinity and specificity [1]. Aptamers have been developed by Larry Gold’s research team at Colorado University in 1990 through systematic evolution of ligands by exponential enrichment (SELEX) method [2]. Various SELEX technologies from the original SELEX to the modified SELEX methods have been developed to screen target-specific aptamers more efficiently [2–5].

To date, a number of aptamers have been screened and displayed great potentials in a wide range of applications, including diagnostic, prognostic, and therapeutic tools in the treatment of human diseases such as cancers and viruses [6]. Aptamers can be largely divided into two types, RNA and DNA aptamers. Both types of aptamers show strong binding affinity for a variety of targets [7–9]. In the early days of aptamer development, RNA aptamers were thought to be superior to DNA aptamers, and research on RNA aptamers was mainly done. Many studies support RNA aptamers display more specific potentials for target specific binding [10–13]. RNA aptamers have a single strand structure with a unique tertiary structure which allows more tight and specific binding to a target. Single strand structured RNA aptamers are usually smaller than DNA aptamers, making it easy to enter cells and beneficially deliver specially designed drugs or additional ligands to the targets either cells or proteins [6]. However, more recently reported studies suggested that when comparing the effect of the DNA aptamer directly with the RNA aptamer, there was no significant difference [14]. Since, the DNA aptamer has also been actively studied. It has a lot of advantages; DNA aptamers do not require transcription and are easier to synthesize than RNA aptamers [14, 15]. The cost and time required for the synthesis of DNA aptamers are less than those of RNA aptamers. These are less reactive and relatively stable due to the C-H bond in the 2′ position of the deoxyribose sugar of the DNA nucleotide. This chemical difference provides the advantage that DNA aptamers are more stable than RNA aptamers. On the other hand, RNA aptamers are chemically unstable because there is a reactive hydroxyl group (−OH) in the 2′ position of the ribose sugar in RNA nucleotides. This -OH group is easily hydrolyzed in alkaline solutions [16]. Nuclease resistance of RNA aptamer has been shown to increase when the 2′-hydroxyl group is removed from the RNA sugar [17, 18].

The high molecular target affinity of aptamers can be used in various directions. One of the most interesting directions is cancer treatment using aptamers. They can be used to diagnose cancer, block cancer signals, or deliver drugs to cancer. The drug delivery System refers to a series of physicochemical techniques that can regulate the delivery and release of pharmaceutically active substances into cells [19]. In this paper, we will discuss the overall content of aptamers used in cancer treatment and the role of aptamers in cancer drug delivery. Specifically, we will focus on how drugs and aptamers can be conjugated and applied to drug delivery for targeted treatment cancers.

## Aptamers

### Antibodies vs aptamers

**Fig. 1:**
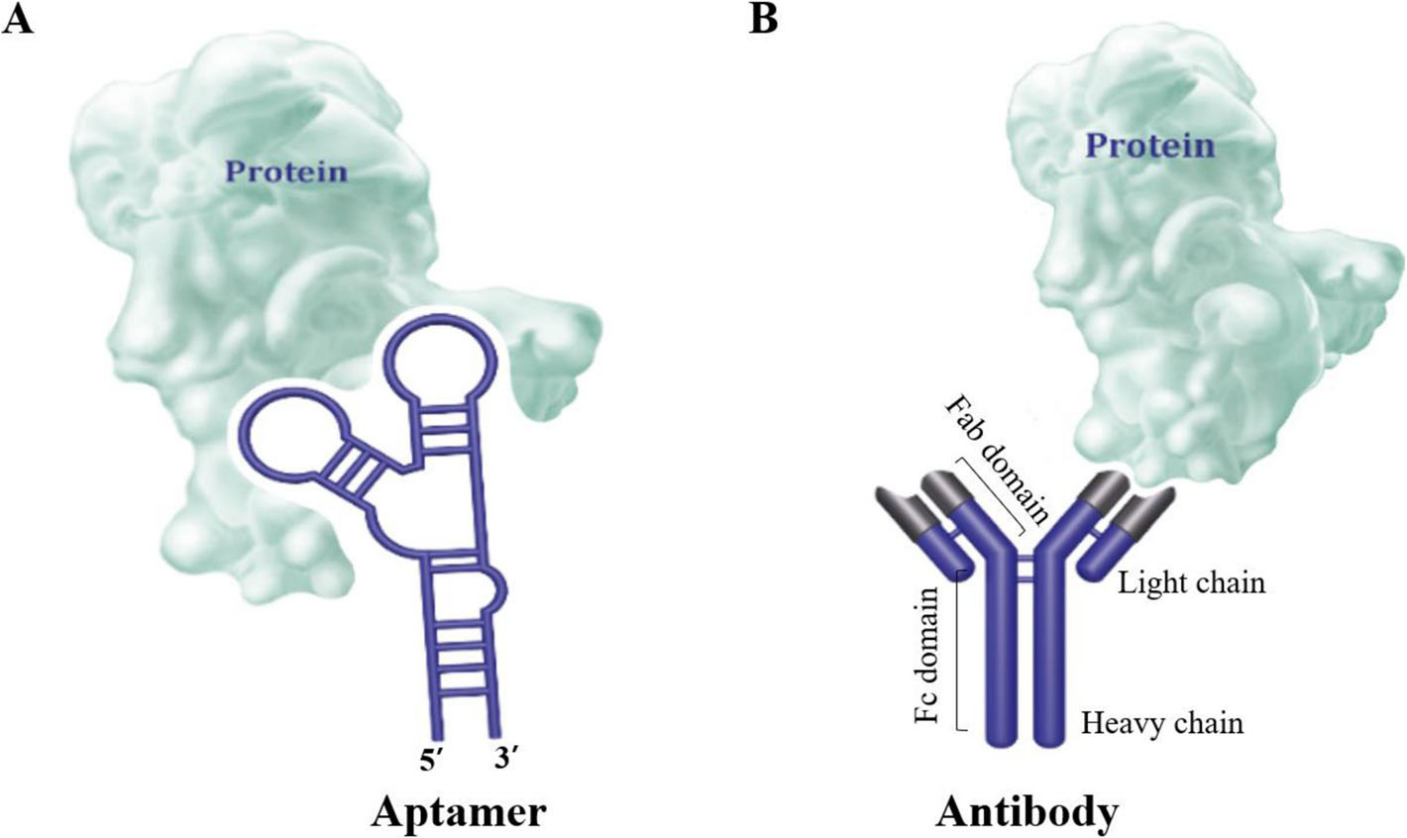
Illustration of how (**A**) aptamer and (**B**) antibody attaches to proteins and structure of (**A**) aptamer and (**B**) antibody

For the clinical development, aptamers have been compared with single antibodies because of their inherent high affinity and specificity that they can bind to target molecules (Fig. 1). Aptamer, regarded as an alternative antibody, has been called “chemical antibody”. Antibody production requires antigens, molecules that are identified as foreign substances and trigger a response by the host’s immune system. This process usually requires several injections of the purified protein [20]. Very small molecules do not elicit an immune response and are therefore excluded from viable targets for antibody production. Since antibodies initially grow in living animals, it is difficult to produce antibodies which targets toxic compounds. Immunization of animals, isolation of antibody-producing cells, and fusion with myeloma cells are essential for the development of monoclonal antibodies. Following this, hybridoma selection and antibody production are also necessary. Each step is time consuming and takes approximately 4–6 months. Antibodies hybridomas are also typically frozen and preserved in liquid nitrogen. Hybridomas can persist over the years, but some are genetically unstable. Hybridoma cells can stop producing antibodies over time because non-producing clones tend to overtake producing clones [21]. On the other hand, aptamers are produced in vitro, they overcome the shortcomings of the above antibodies and can select a wider range of targets (Table 1). Traditional aptamer selection is typically an in vitro process that completes within two to three months and the transformation process completes much faster. In terms of size, the aptamers have more advantages than the antibodies. Typically, IgG antibodies are 150–170 kDa, whereas aptamers with 30 to 80 nucleotides have 12–30 kDa of molecular weight (Table 1). The large size antibody shows the limited membrane permeability, especially, when targeting dense tissues [22, 23]. On the contrary, aptamers have demonstrated an improved access to tissues in in vivo imaging due to their small size [24]. Some aptamers and aptamer complexes get into the cytosol area through cell membranes and even pass-through blood-brain barriers. One of the most important drawbacks of therapeutic antibodies is their immunogenicity, which elicits an immune response to the therapeutic antibodies and produces counter-antibodies, which ultimately reduces the efficacy of antibody therapy. These counter antibodies (so-called as anti-drug antibodies) can adversely affect treatment efficacy by inducing unwanted reactions or reducing activity to drugs. Various factors, including drug impurities, excipients, dosage therapy, disease types and stages, target cell types, and combination therapy can affect immunogenicity. Pfizer’s latest study found that human/humanized antibody drugs targeting T cells have a high incidence of anti-drug antibodies in 27% of patients. However, aptamers are essentially non-immunogenic, as have been demonstrated in recent biomedical studies [24, 25] and suitable for in vivo use without extensive modification. Since aptamers are chemically synthesized, they can be easily maintained, besides reproduction is always reliable with information on the aptamer sequence and all modifications (Table 1). In addition, low cost and ease of modification can be the advantages of aptamers against antibodies. Due to these many advantages, aptamers are emerging as future target treatment candidates to replace antibodies.

**Table 1:** The difference between aptamers and antibodies

	Aptamers	Antibodies
Size	small (~ 12–30 kDa)	relatively big (~ 150 – 170 kDa)
Target	wide range	immune related protein
Synthesis	simple (chemically synthesis)	complicate (in vivo production)
Stability	stable	susceptible to temperature and pH
Modification	various modification	limited modification
Storage term	long	relatively short
Cost	low	high
Immunogenicity	low	high

### Aptamers for cancer therapy

Cancer biomarkers are molecules that represent abnormal conditions in cancer and play important roles in many biological processes, including cell migration, cell proliferation, signal transduction, and cell-cell interactions. Many studies have identified molecules such as membrane proteins, transcription factors, and growth factors as good biomarkers. The well-characterized membrane proteins, especially endogenously overexpressed on the surface of cancer cells, are potential targets for cancer treatments. Aptamers are sensitive to the detection of these cancer biomarkers and have emerged as new target materials due to their high affinity to target molecules. They recognize and bind the target through the formation of a voluntary three-dimensional structure with the aim of improving therapeutic effects and reducing unnecessary toxicity to non-cancer cells. The main challenge is to identify aptamer sequences that bind to specific biomarkers in cancer cells. Over the past a few decades, many aptamers have been developed specific to tumor-related biomarkers and have been extensively studied for the therapeutic applications against cancers, such as breast cancer, colon rectal adenocarcinoma, lung cancer, and prostate cancer. In particular, numerous aptamers have been established targeting cancer-specific signature markers such as immunosuppressive programmed cell death protein 1 (PD-1), immunosuppressive CD137, CD134, tumor-forming platelet-derived growth factor, and vascular endothelial growth factor [26]. Aptamers can act as antagonists or agonists respectively that inhibit or stimulate the interaction of tumor-associated targets [27]. Induction and maintenance of immune responses is strictly regulated by continuous immune check points. Currently, the most relevant immunological checkpoints in cancer immunotherapy are cytotoxic T- lymphocyte-associated protein 4 (CTLA-4) and programmed cell death protein 1 (PD-1) expressed in T lymphocytes and programmed cell death ligand 1 (PD-L1) expressed on the cancer surface. Many researchers are working to effectively block these immune checkpoints with aptamers, and quite a few aptamers have been studied. Because aptamers exhibit remarkable affinity and specificity in ligand targeting, they can be used to stimulate or inhibit targets of interest such as receptors and growth factors responsible for cancer progression. In 2003, Gilboa’s group announced the development of the first immune checkpoint blocking RNA aptamer that binds to CTLA-4. CTLA-4 engagements increase the threshold of the signal required for T-cell activation, delivering negative signals that attenuate T-cell responses. Blocking the CTLA-4 signal is consistent with observations that T cell receptors are upregulated and CD28-dependent proliferation of T cells occurs. This selection of anti-CTLA-4 aptamers was first used for immunotherapeutic purposes and opened the door to new platforms [28]. Furthermore, several aptamers have been described in the context of immunotherapy for some cytokine blockades. Aptamers, known as R5A1 binding to IL-10R, were selected and optimized to block interactions between IL-10 and its receptors on the cell surface of the immune system. IL-10 is secreted by tumor cells and is known to promote immunomodulatory responses favorable to tumor formation and growth [29]. Activating the positive signal corresponding to tapping the accelerator has been one of the key strategies in cancer immunotherapy. In addition to activation of signals due to simultaneous binding of CD3, MHC-I, and TCR known as second co-stimulation signals are required for T cells to be properly activated. The tumor microenvironment typically lacks co-stimulating ligands such as CD80 or CD86. The lack of these co-stimulations makes CD8 + T cells paralyzed and unable to trigger an immune response. Several aptamers selected for the main co-stimulating receptor (4-1BB, OX-40, or CD28) were selected and manipulated to co-stimulate T cells. Clinically famous aptamer in anticancer studies is AS1411, a 26-nucleotide guanosine-rich DNA sequence that specifically binds to overexpressed or a potential nucleolin in many types of cancer cells. In addition to its cancer-targeting efficacy, AS1411 prevents nucleolin from binding to Bcl-2 tumor genes, inhibiting cell escape from apoptosis. Several preclinical studies of very low concentration AS1411-junction nanosystem has shown significant inhibitory effects on various tumor cell lines. Inspired by the preclinical success of AS1411, the commercial version (created in Aptamera Inc., KY, Louisville) has passed clinical trials for the effects of anti-acute myeloid leukemia (AML) and renal cell carcinoma. Another well-known aptamer capable of potential clinical applications is the A10 which specifically binds to prostate cancer biomarkers, prostate specific membrane antigens (PSMAs). Many studies have demonstrated significant effects such as specific in vivo therapeutic efficacy against prostate cancer in PSMA-expressed Lymph node Carcinoma of the Prostate cell xenograft mice models. As one type of cancer therapy using aptamer, targeted molecular imaging using aptamers is an active research field. For target imaging, aptamer has demonstrated cancer-specific recognition in tissue imaging and in vivo imaging in several cancer models. For in vivo use, DNA aptamers (anti-MUC1, anti-nucleotide, anti-EGFRvIII, anti-cancer cell type-specific aptamer) and RNA aptamers (anti-PSMA aptamers) are used for cancer-specific detection using a variety of imaging. In that sense, it can be seen as suitable for preclinical phase technology (e.g., fluorescence, PET, SPECT, MRI, CT, and US). This suggests that aptamers have great potential for target video topics to distinguish disease-specific targets which can be applied to clinics. Every imaging technique has its own detection mechanism and unique advantages and disadvantages. With the use of such various aptamers, studies to help cancer treatment are actively underway, and there are many aptamers that have been conducted up to clinical trials with good results. In addition to the therapy used only by aptamers, aptamers can be used to specifically deliver other treatments, including small interference RNA (siRNA), microRNA (miRNA), short hairpin RNA (shRNA), and chemotherapy. A study was conducted in 2006 by Paloma H Giangrande research team, which confirmed the antitumor activity in a prostate cancer by knock down PLK1 by conjugating PK1 siRNA to A10 aptamer [30]. Various aptamers specific to cancer biomarkers have been used to deliver treatments with increased local concentrations and therapeutic efficacy. Due to its various advantages: stability for high storage, ease of synthesis and functionalization, and ease of developing low immunogenicity and antidote, aptamers have been widely used in the treatment and diagnosis of targeted cancers [31].

## Aptamer-drug conjugation chemistry

Aptamers are promising candidates for application in targeted therapy due to their characteristics. Their high specificity and selectivity make them excellent candidates for targeted delivery of therapeutic agents [32]. Those can be developed against toxins or hypoimmunogenic agents that exceed the capabilities of current antibody development techniques and can be used as carriers for the aptamer-drug conjugate (ApDC)-based targeted drug delivery. These advantages make aptamers attractive for ApDC development for targeted therapy. As another major type of recognition ligand, antibodies have been studied extensively in the last decades to develop the antibody-drug conjugates (ADCs) [33] [34]. The general stability and structural reversibility of aptamers enables to design various types of ApDC. Similar to ADC, ApDC is usually made up of three molecular parts: ligand (aptamer), linker, and warhead (drug). Aptamers act as recognition ligands that target disease sites and/or guide the delivery of therapeutic agents that modulate the biological function of the target biomarker. Chemical stability, simplicity of chemical modification, and ready-made molecular engineering of aptamers enables easy and programmable conjugation with a wide range of therapeutics [35, 36]. In this regard, the provision of ApDC by bonding the cytotoxic payload to the aptamer has been proven to be effective in inhibiting tumor growth in vitro and in vivo [37].

### Construction of ApDC using nucleotide analogs

**Fig. 2:**
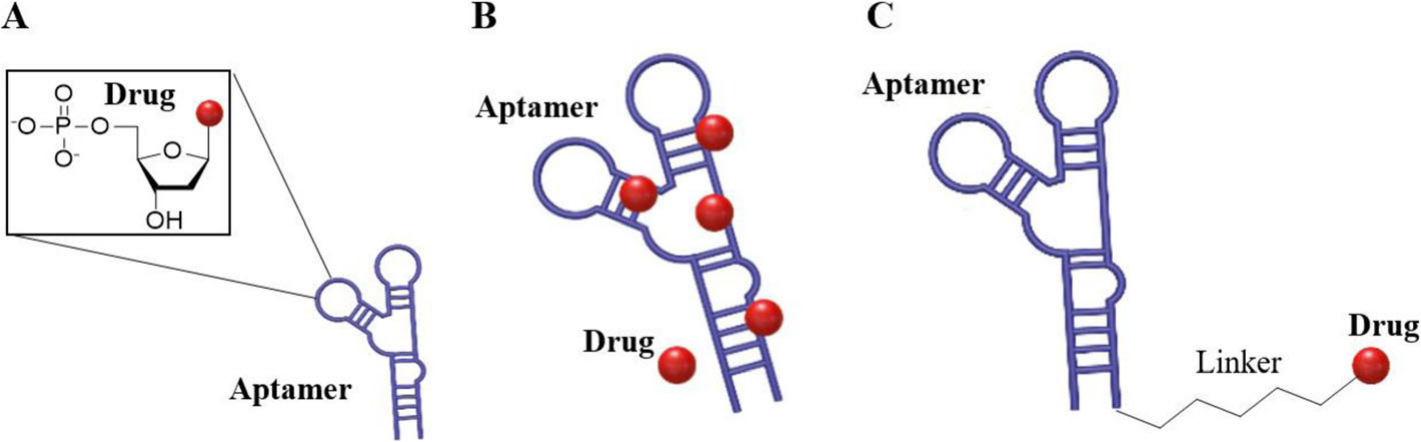
(**A**) ApDC constructed by Nucleotide Analogs. (**B**) ApDC by drug and aptamer intercalation (**C**) ApDC by using linker between drug and an aptamer

Nucleotides contain a sugar, nitrogenous bases, and phosphate group with one to three phosphates. Nucleotide analogs can be used in therapeutic drugs. They are among the first chemotherapy agents introduced for cancer treatment. The compound family has grown to include a variety of purine and pyrimidine derivatives that have activity in both solid tumors and malignant diseases in the blood. The drug acts as a metabolic antagonist, competes with physiological nucleic acids, and interacts with many intracellular targets to induce cytotoxicity. Recently, the identification and characterization of nucleotides transporter and nucleotides metabolism are underway. It also provides an opportunity to improve the anticancer effect by increasing understanding of the molecular mechanisms of anticancer nucleotides activity. The strategy to optimize intracellular analog accumulation and improve cancer cell selectivity has been proven to be beneficial in clinical trials. Nucleotide analogs and nucleobases are pharmacologically diverse families, including: cytotoxic compounds, antiviral agents, and immunosuppressive molecules [11] [38]. This nucleotide analogs to nucleic acid-based aptamer can be applied. ApDC nucleotide analogs can be formed by inserting nucleotide analogs between aptamer sequences, which are originally known as anticancer mechanisms (Fig. 2A). Therefore, both the structural anticancer effect of the aptamer and the chemotherapeutic effect of the nucleotide analog can be utilized [39].

### ApDC by drug intercalation

The intercalation is the reversibly inclusion or insertion of molecules (or ions) into layer materials with layer structures. In biochemistry, intercalation techniques are applied for inserting molecules between DNA bases and can be used analyzing DNA. Mainwaring and others defined DNA intercalation as inserting molecules between two adjacent pairs of bases in the DNA double helix. The intercalating ligand is characterized by the possession of an extended electron deficient planar directional ring system. Upon binding, they expand and unroll the deoxyribose-phosphate backbone and are stabilized by – lamination interaction with the plane aromatic base. Intercalation also results in fluid dynamic changes in DNA due to a reduction in torsion between base pair layers, lengthening of DNA itself, rigidity of the helix, and mass reduction per unit length. This effect can be completely reversed when the intercalator is removed unless the DNA dual structure is destroyed by the removal process. In short, the dsDNA structure separates into two single strands by intercalating environment changes entering the cells and releases the intercalated drugs [40] [41].. Strategies using this DNA intercalation for aptamers are being studied for ApDCs. It has the advantage of being able to easily conjugation aptamers and drugs without going through a complicated chemical transformation process or synthesis process, and there are many studies using various chemical drugs including doxorubicin (Dox) and various fluorescent dyes represented by quinoline blue [26, 42] (Fig. 2B).

### ApDCs by using chemical linker

Covalent drug binding has been extensively explored to develop ApDCs with more potential for site-specific modifications; for this other linker chemicals such as enzyme reactions, pH-labile dynamics, temperature dependents, and non-cleavable linkers have been developed. Chemical linkers have also been widely applied to the construction of the ApDC conjugate (Fig. 2C). The most used ApDC is generated by the linker method. These linkers have cleavable function and mainly have used in antibody drug conjugation (ADC). There are three types of methods that usually applied to attach a linker to an aptamer. The first is hydrazone linker. Hydrazone is associated with ketones and aldehydes by replacing oxygen with NHH2 functional groups. These are usually formed by the action of hydrazine on ketones or aldehydes. Hydrazone group which is unstable in acid is decomposed through hydrolysis when aptamer and drug by ApDC being transported to acidic endosomes and lysosomes, and releases the drug [43]. The second is thiol-related chemistry. Michael addition of thiol-to-thiol maleimide binding chemistry or to maleimide or Michael receptors is commonly used on the surface of a target ligand or nanoparticle drug delivery system for bioconjugation or macromolecules of thiolated (−SH) drugs. A drug can be thiolated and used with attached a thiol group. Also, a thiol group can be attached to an aptamer. A disulfide bond is made by reacting a thiolated drug with an aptamer. A thiolated PEG or maleimide also can be used between the drug and aptamers to increase the stability of the compound. The last is the linker that can be cleaved by Cathepsin B which is a lysosome protease that is overexpressed in various cancer cells and is involved in numerous carcinogenesis processes in humans. Cathepsin B has a relatively wide range of substrates. However, specific sequences such as phenylalanine-lysine (Phe-Lys) and valine-citruline (Val-Cit) are recognized first and peptides are bound and cut at the C-terminal side of these sequences. Upon endocytosis and transport to the lysosome, cathepsin B selectively cleaves this linker and is released without cytotoxicity.

## Aptamer chemical drug conjugates for cancer therapy

Various ApDcs have been studied worldwide. What kind of aptamer and drug conjugation studies have been conducted based on the aptamer type in the cancer treatment will be discussed (Table 2).

**Table 2:** Characters of aptamers conjugated with drugs

Aptamer	Type	Target	Conjugated drug	References
P19	RNA	PDAC	Monomethyl auristatin E, 5-fluorouracil, gemcitabine	Yoo et al. (2017), Sora et al. (2016)
Sgc8	DNA	PTK7	Doxorubicin	Yu-Fen et al.(2009)
AS1411	DNA	Nucleolin	Doxorubicin, paclitaxel, triptolide	Thu et al. (2015), Fangfei et al. (2017)
A10	RNA	PSMA	Doxorubicin,	Vaishali et al. (2006)
Her2 aptamer	RNA	Her2	DM1(mertansine)	Hwa et al. (2020)

### P19 aptamer

**Fig. 3:**
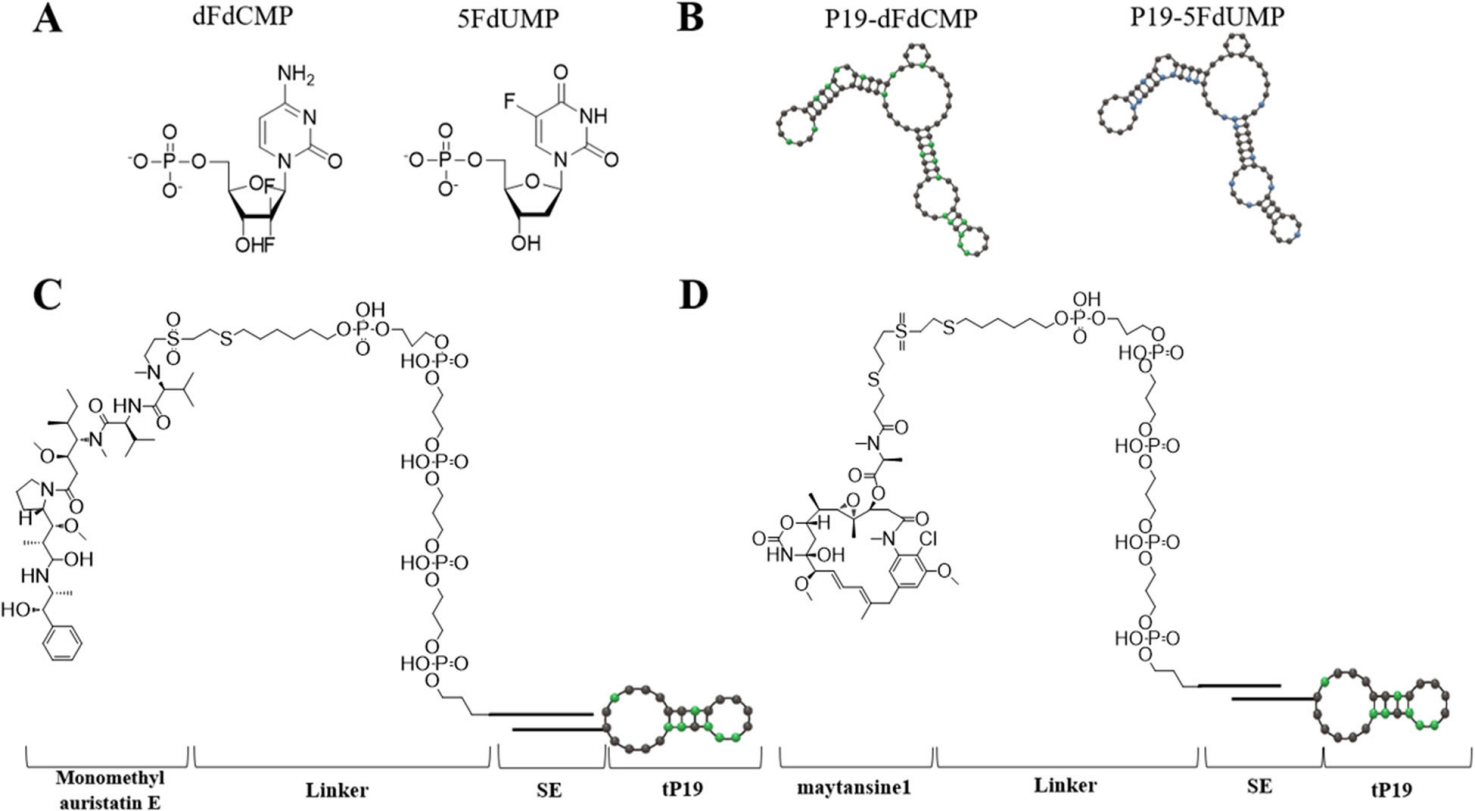
(**A**) Chemical structure of gemcitabine monophosphate (dFdCMP) and 5-Fluorouracil monophosphate (5FdUMP), (**B**) dFdCMP (P19-dFdCMP) and 5FdUMP (P19–5FdUM) conjugated structure with P19 is indicated by a red dot representing dFdCMP and a blue dot representing 5FdUMP, (**C**) Chemical structure of MMAE (monomethyl auristatin E) conjugated with P19 by Linker and SE (Sticky sequence), (**D**) Chemical structure of DM1(maytansine 1) conjugated with P19 by linker and SE (Sticky sequence)

The P19 aptamer is an RNA aptamer selected by the John *J. Rossi* group in panc-1 [44]. They used a 2’F-RNA combinatorial library to isolate the 2’F RNA aptamer (P19) for pancreatic ductal adenocarcinoma target delivery via whole cell-based SELEX. These group conjugated P19 to a derivative of gemcitabine and 5-fluorouracil (5-FU), or monomethyl auristatin E (MMAE) and maytansine 1 (DM1) [45]. P19-gemcitabine and P19–5-FU phosphorylated histone H2AX for Ser139 (ɤ-H2AX) that is a biomarker of DNA double strand breaks (DSBs) and inhibited cell proliferation in PANC-1, gemcitabine-resistant pancreatic cancer cells. In addition, P19-MMAE and P19-DM1 affected mitotic cells. It caused G2/M phase arrest and inhibited cell proliferation. Moreover, the cytotoxicity of P19-MMAE and P19-DM1 in normal cells was minimal in the human breast cancer cell line MCF7. Gemcitabine and 5-fluorouracil were conjugated by forming a nucleotide analog to the P19 aptamer and MMAE and DM1 were conjugated using a linker. A phosphate group was added to gemcitabine to make gemcitabine monophosphate (dFdCMP), and a phosphate group was also added to 5-FU to make 5-Fluorouracil monophosphate (5FdUMP) (Fig. 3A). dFdCMP and 5FdUMP with gemcitabine or 5-fluorouracil attached were added to the synthesis of P19 RNA aptamer to make gemcitabine conjugated P19 or 5-fluorouracil conjugated P19 (Fig. 3B). They found that when P19-dFdCMP and P19–5FdUMP were treated to pancreatic cancer more DNA double strands breaks occurring than when only P19 was treated. Eventually, more double strand breaks caused by ApDC led to lower cell proliferation of pancreatic cancer cells. A carbon linker was used for the conjugation of P19 with MMAE and DM1 using a linker (Fig. 3C and D). First, the entire length of P19 was truncated into smaller 27-mer units (tP19) to facilitate increased binding affinity and allow large-scale chemical synthesis. Next, 50 ends were attached to MMAE or DM1 via a sticky sequence (SE) to prevent structural disruption of tP19. After that, tP19-MMAE and tP19-DM1 complexes were created by attaching tP19-SE linked to MMAE-SE or DM1-SE in a buffer for ApDC assembly. Treatment with tP19, the cell cycle was not disturbed at any stage. However, treatment with tP19-MMAE and tP19-DM1 significantly increased the number of cells entering the G2/M phase. In addition, in cell proliferation, both tP19-MMAE and tP19-DM1 treated cancer cells were induced significant inhibition.

### Sgc8c aptamer

**Fig. 4:**
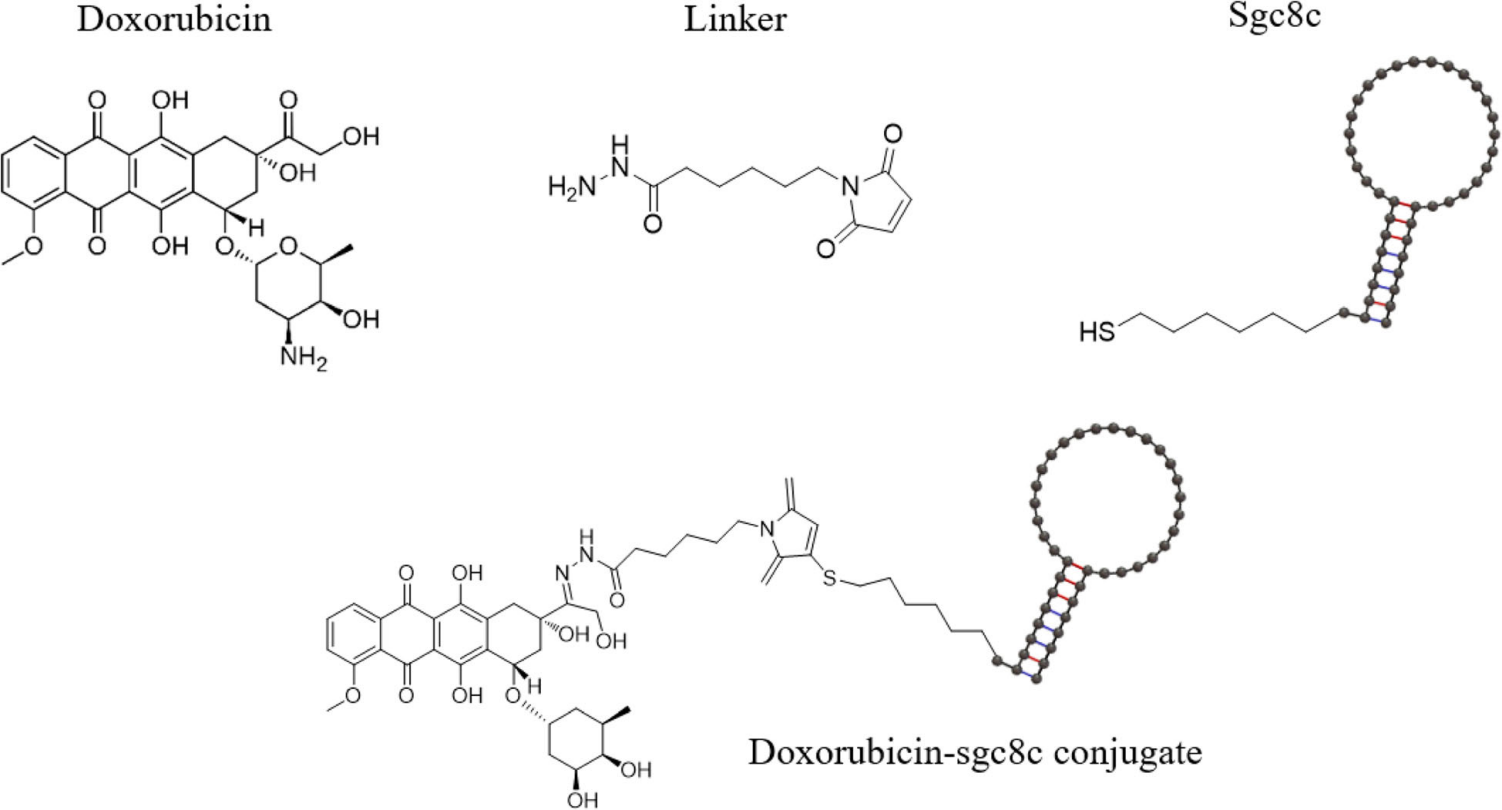
Schematic model of synthesizing Sgc8c conjugated with Dox (doxorubicin) by linker for targeting leukemia cells

Sgc8 was selected from CCRF-CEM cell line of human acute lymphoblastic leukemia (ALL) [46]. It was specifically bound to many leukemia cells, including most T cell acute lymphoblastic leukemia (T-ALL) cells and acute myeloid leukemia (AML) cells, as well as some B-cell acute lymphoblastic leukemia (BALL) cells. It showed high affinity and specificity to the cell surface protein or membrane [47]. In addition, various modifications were attempted to make sgc8 smaller for ease of handling, and several truncated Sgc8 sequences (sgc8a, sgc8b, sgc8c, sgc8d) were synthesized. As a result of measuring the Kd value, it was confirmed that only the sgc8c sequence was very close to the Kd (~ 0.78 nM) of the full length sgc8, and that the binding affinity to the target was not lost [48]. A hydrazone linker to bond sgc8c to Dox were selected to release a chemical agent from the conjugate after internalization (Fig. 4A). In several studies, Dox C-13 hydrazone derivatives had a cytotoxic effect similar to non-conjugated Dox [49] and have already demonstrated that Dox can be released at pH 4.5 to 5.5 [50]. Less fluorescence was detected in CCRF-CEM cells saturated with sgc8c-Dox, confirming that the binding between sgc8c-Dox conjugates to CCRFCEM cells was through specific binding of sgc8c to its target. In addition, the binding affinity (Kd) of sgc8c-Dox was confirmed to be 2.0 ± 0.2 nM by indirect fluorescence. These results were very similar to those without Dox conjugation, sgc8c. These were recognized that the aptamer-Dox conjugate maintained a specific binding and high affinity for targeted cancer cells. It was also observed that sgc8c-Dox conjugate had a 6.7-fold increase in toxicity to the target CCRF-CEM cells compared to other cells using MTT assay [51].

### AS1411 aptamer

**Fig. 5:**
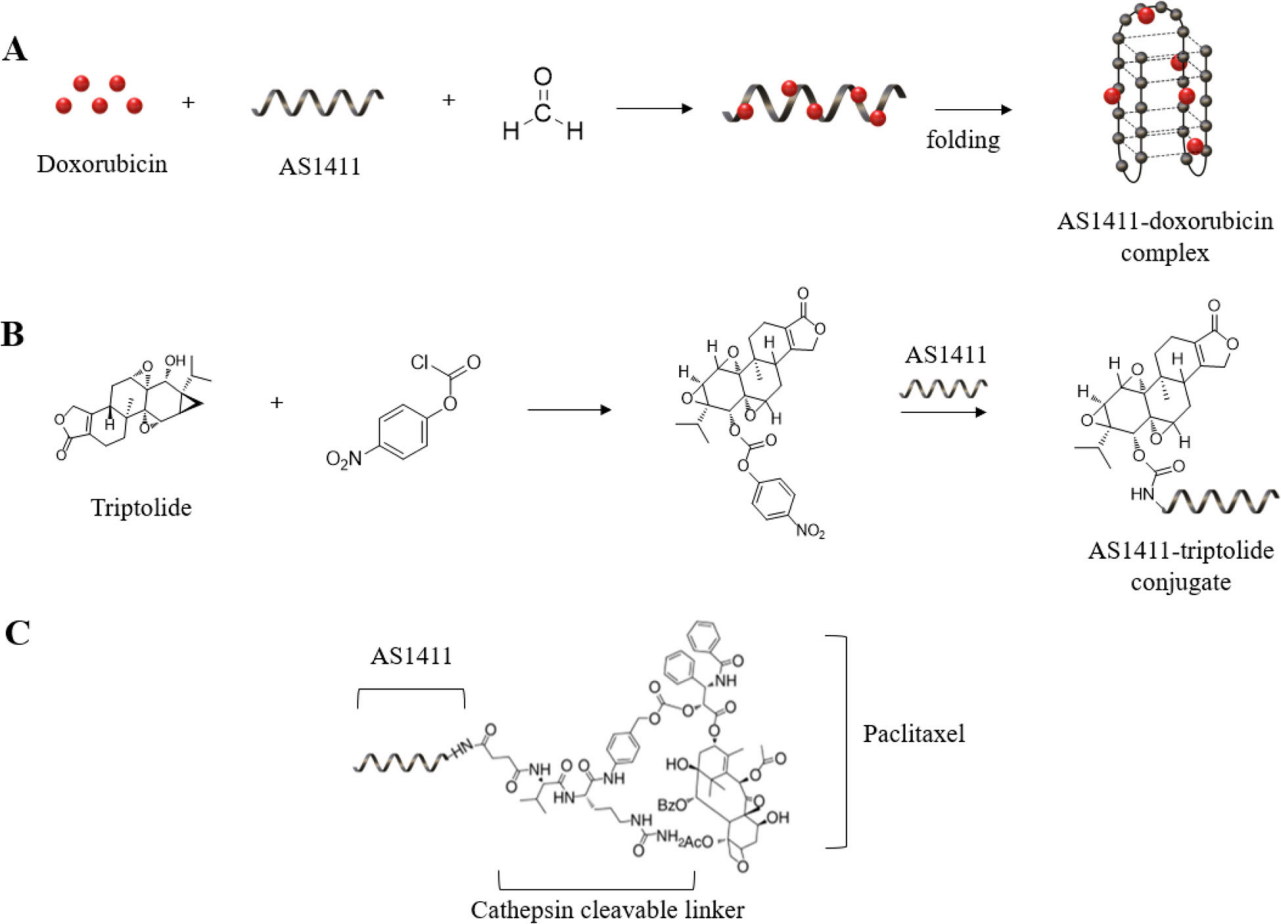
(**A**) Schematic model of synthesizing Dox-AS1411 complex, (**B**) Schematic model of synthesizing NCP-TP and triplolide AS1411 conjugation, NPC-TP = nitro phenyl chloroformyl – triplolide (**C**) Chemical structure of paclitaxel conjugated with AS1411 by cathepsin-B cleavable linker

AS1411 (AGRO100) is a well-known DNA aptamer for the treatment of AML and renal cell carcinoma that have passed clinical trials [52, 53]. Several studies have shown that AS1411 induces apoptosis in tumor cells by binding to plasma membrane nucleolin which is highly expressed in many cancer cells [54–56]. Although the effectiveness of AS1411 has been evaluated in many cancers, this aptamer has limited therapeutic effects. Those limitations are serum instability, renal filtration, and endocytic escape and other restrictions more specific to aptamer include nuclease sensitivity and aptamer specificity [57].. To solve this problem, conjugation of various drugs and AS1411 has been attempted. First, AS1411-Dox complex (AS1411-Dox) that combines the targeting ability of AS1411 with the therapeutic efficacy of Dox has developed [58]. Dox can bind to DNA by intercalation [51] [59]. Dox adducts were incubated with formaldehyde and manufactured, while AS1411-Dox adducts were produced by incubating aptamers with Dox and formaldehyde in a reaction buffer solution for one night at 10 °C (Fig. 5A). AS1411-Dox adduct efficiently targeted liver cancer cells in vitro, delivered Dox to target cells, and reduced cell viability to a level comparable to that of free Dox. AS1411-Dox adduct inhibited tumor growth and induced tumor cell death by targeting tumor tissue with high efficiency without inducing apoptosis in non-tumor tissues. Second, AS1411 was conjugated with triptolide [60], which has been utilized as an effective therapeutic agent for various types of cancer treatment [61] as a combinational therapy with aptamer. The AS1411-triptolide conjugate was synthesized via hydrazone linker-like C-N bond formation. That conjugation worked by incubating amino-AS1411 for 1 h with p-nitro phenyl format triptolide (NPC-TP) (Fig. 5B). AS1411-triptolide conjugate not only showed superior stability compared to AS1411 but was also able to specifically recognize cancer cells. Aptamer treatment significantly improved the antitumor effect of triptolide in vivo and could be widely applied in many cancers overexpressing nucleolin. Third, nucleolin aptamer-paclitaxel conjugate (NucA-PTX) was designed and synthesized to selectively transfer PTX to tumor site [62]. The tumor-targeted AS1411 was bonded to paclitaxel via a cathepsin B-cuttable linker and NucA connected to PTX at C-2’s position (Fig. 5C). NucA-PTX made it possible to effectively deliver paclitaxel into cells and showed a much more effective anticancer effect than only paclitaxel or only AS1411 in vivo and in vitro without any specific toxicity in the body.

### A10 aptamer

**Fig. 6:**
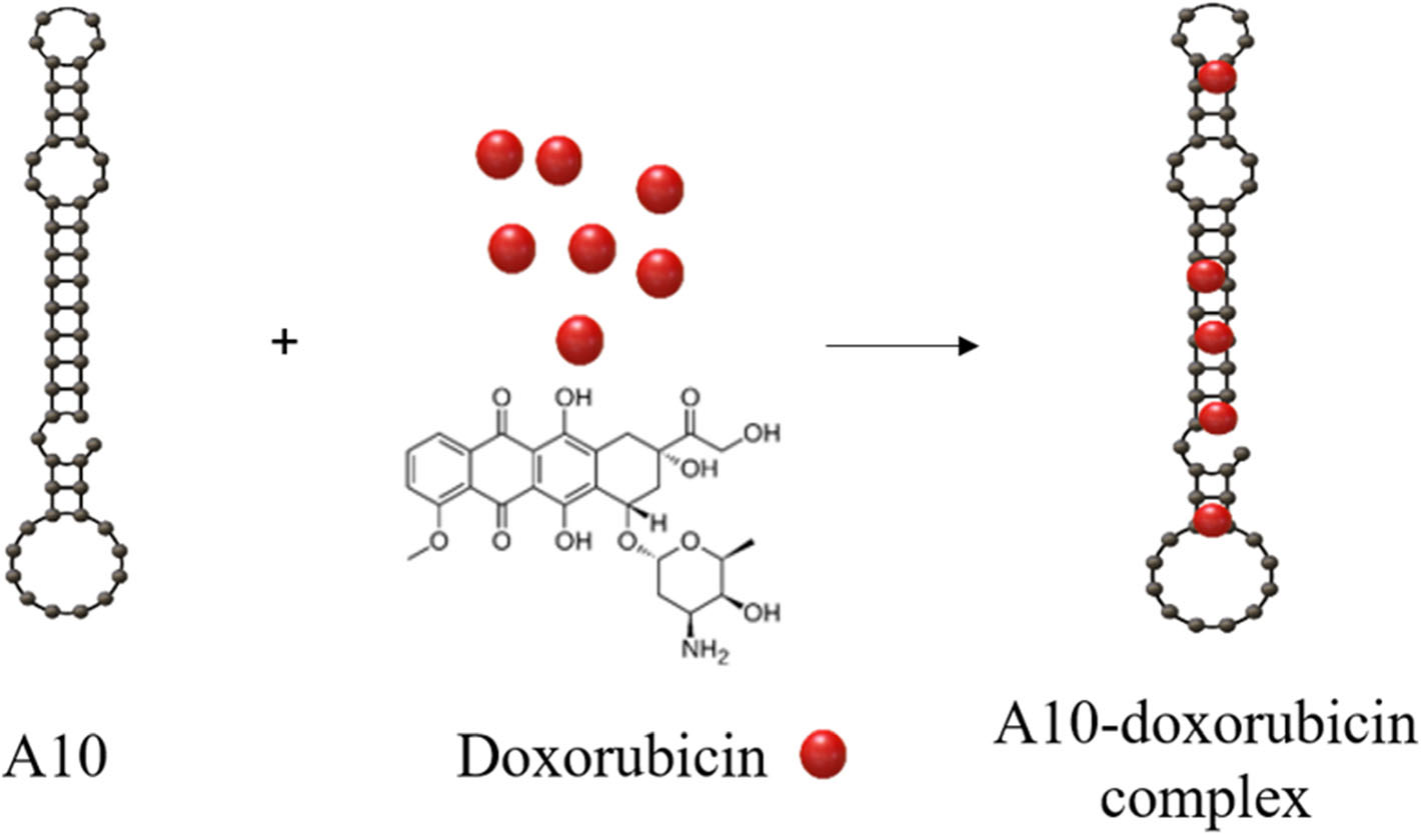
Schematic model of how to make physical intercalation complex between aptamer and drug using characteristic of drug

A10, 2′-fluoropyrimidine RNA aptamer binds to PSMA with high affinity and specificity. As shown above, Dox is known to preferentially bind to the double strand 5′-GC-3 ‘or 5’-CG-3′ sequence through intercalation, and one possible structure is the intercalation of Dox to the A10 when evaluating the predicted A10’s aptamer secondary structure [63] (Fig. 6A). The incubation of Dox and A10 aptamers resulted in the maximum extinction of Dox fluorescence at about 1:1.2 mol equivalents of Dox and App tamers, suggesting that Dox inserts into the predicted CG sequence to produce physical complex with the A10 aptamer. The ability to maintain aptamer binding properties while Dox is inserted into it allows for targeted delivery of Dox to cells expressing aptamer targets. The aptamer-Dox physical complexes also confirmed stability and higher target activity in cell culture mediums, and growth inhibition analysis (MTT analysis) showed that the aptamer-Dox physical complex releases Dox molecules within lymph node carcinoma of the prostate cells after infusion and kills PSMA-positive cancer cells more efficiently.

### HER2 RNA aptamer

**Fig. 7:**
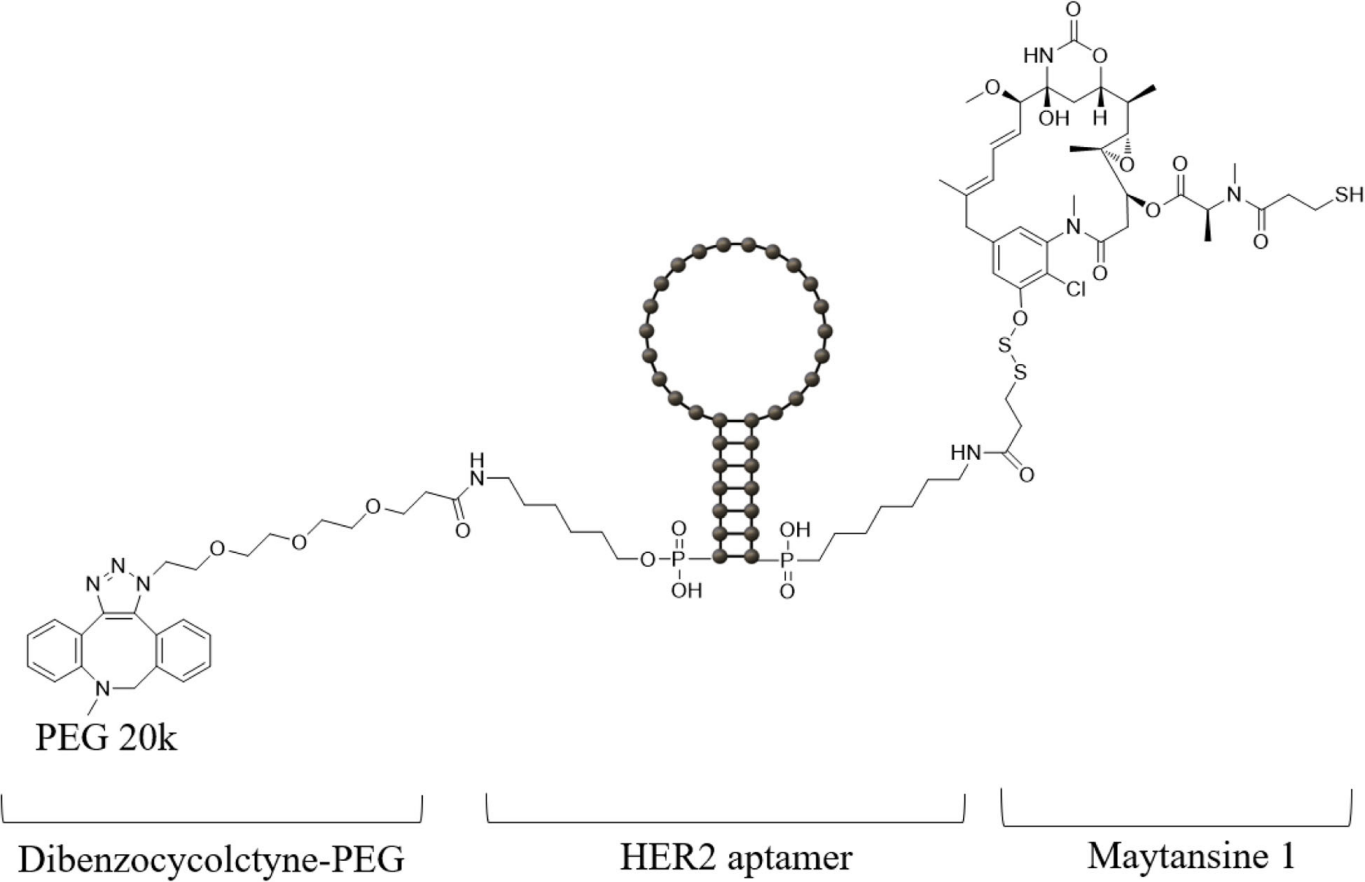
Chemical structure of DM1(maytansine1) that conjugated with HER2 aptamer combined with PEG 20 k (Polyethyleneglycol 20 k)

Sung-Chun Kim’ team (Biois Co. Ltd’s) synthesized HER2 specific RNA aptamers that bind efficiently to HER-positive BT-474 breast cancer cells but not to HER2-negative MDA-MB-231 breast cancer cells [64]. They conjugated the anticancer DM1 to a HER2-specific aptamer using a cleavable disulfide linker. In addition, polyethylene glycol polymer was also conjugated to the aptamer end for long-term circulation in vivo. HER2 RNA aptamer modified by pyrimidine fluorination showed increased stability compared to the unmodified RNA aptamer [65] (Fig. 7A). For the synthesis of the HER2 ApDC, a 20 kDa PEG molecule was attached to the 5′ end of the HER2 RNA aptamer and a DM1 molecule to the other 3′ end (Fig. 2A). DM1 is a widely used anticancer agent and because it is the thiol form of mertansine, it has a structural advantage in binding to a carrier molecule (Fig. 7B). Furthermore, PEGylation of aptamers at the 5′ end can prevent renal clearance, enhancing the half-life of circulating ApDCs. DM1-HER2 aptamer had a cytotoxic effect on HER2 positive breast cancer in vitro, and free HER2 aptamer had no effect, thus it was confirmed that DM1-HER2 aptamer had a cytotoxic effect by DM1. Cancer was more effectively treated by DM1-HER2 aptamer compared to only DM1 or HER2 aptamer in vivo. In addition, in histological examination, when DM1-HER2 aptamer was treated compared to DM1, the inflammatory response in the kidneys and spleen was significantly lowered, and body toxicity was also reduced.

## Conclusion

Aptamers have developed and used for various cancer treatments due to their high affinity to target molecules and improving therapeutic effects and reducing unnecessary toxicity to non-cancer cells. Anti-CTLA-4 aptamer, R5A1IL-10R binding aptamer, and 4-1BB aptamer have used for immunotherapeutic purposes to treat cancers. In addition to the therapy used only by aptamers, aptamers can be used to specifically deliver other treatments, including small interference RNA (siRNA), microRNA (miRNA), short hairpin RNA (shRNA), and chemotherapy. The general chemical and thermal stability and structural reversibility enable various designs of ApDC which is usually made up of three molecular parts: ligand (aptamer), linker, and warhead (drug). Aptamers act as recognition ligands that target disease sites and/or guide the delivery of therapeutic agents that modulate the biological function of the target biomarker. Three types of ApDC were reviewed how aptamer and drug conjugated in the chemistry. In addition, research results of several aptamer and drug conjugated, ApDCs such as P19-dFdCMP, P19–5FdUMP, sgc8c-Dox, and NucA-PTX are reviewed. Due to the advanced technology of cancer drug delivery and combination technologies, aptamer and ApDCs now become an efficient means for targeted cancer treatment that reduces potential toxicity and increases therapeutic efficacy.

## Data Availability

Not applicable.

## Abbreviations

ApDC: Aptamer drug conjugate
SELEX: systematic evolution of ligands by exponential enrichment
CTLA-4: cytotoxic T- lymphocyte-associated protein 4
AML: acute myeloid leukemia
ADC: antibody-drug conjugate
5-FU: 5-fluorouracil
MMAE: monomethyl auristatin E
DM1: maytansine 1
dFdCMP: gemcitabine monophosphate
5FdUMP: 5-FU monophosphate
Dox: Doxorubicin
NucA: nucleolin aptamer
PTX: paclitaxel
PSMA: prostate specific membrane antigen

## References

[1] Ali MH, Elsherbiny ME, Emara M (2019). Updates on Aptamer Research. Int J Mol Sci.

[2] Ellington AD, Szostak JW (1990). In vitro selection of RNA molecules that bind specific ligands. Nature.

[3] Tuerk C, Gold L (1990). Systematic evolution of ligands by exponential enrichment: RNA ligands to bacteriophage T4 DNA polymerase. Science.

[4] Ellington AD, Szostak JW (1992). Selection in vitro of single-stranded DNA molecules that fold into specific ligand-binding structures. Nature.

[5] Bock LC, Griffin LC, Latham JA, Vermaas EH, Toole JJ (1992). Selection of single-stranded DNA molecules that bind and inhibit human thrombin. Nature.

[6] Germer K, Leonard M, Zhang X (2013). RNA aptamers and their therapeutic and diagnostic applications. Int J Biochem Mol Biol.

[7] Neves MA, Reinstein O, Saad M, Johnson PE (2010). Defining the secondary structural requirements of a cocaine-binding aptamer by a thermodynamic and mutation study. Biophys Chem.

[8] Baugh C, Grate D, Wilson C (2000). 2.8 a crystal structure of the malachite green aptamer. J Mol Biol.

[9] Thorsten Dieckmann EF, Xhao X, Szostak J, Feigon J. Structural investigations of RNA and DNA aptamers in solution. J Cell Biochem. 1995.

[10] Que-Gewirth NS, Sullenger BA (2007). Gene therapy progress and prospects: RNA aptamers. Gene Ther.

[11] Keefe AD, Pai S, Ellington A (2010). Aptamers as therapeutics. Nat Rev Drug Discov.

[12] Thiel KW, Giangrande PH (2010). Intracellular delivery of RNA-based therapeutics using aptamers. Ther Deliv.

[13] Guo P (2010). The emerging field of RNA nanotechnology. Nat Nanotechnol.

[14] Hernandez LI, Machado I, Schafer T, Hernandez FJ (2015). Aptamers overview: selection, features and applications. Curr Top Med Chem.

[15] Wilson DS, Szostak JW (1999). In vitro selection of functional nucleic acids. Annu Rev Biochem.

[16] Barciszewski J, Clark BFC (2012). RNA biochemistry and biotechnology.

[17] Adler A, Forster N, Homann M, Göringer HU (2008). Post-SELEX chemical optimization of a trypanosome-specific RNA aptamer. Comb Chem High Throughput Screen.

[18] Wilson C, Keefe AD (2006). Building oligonucleotide therapeutics using non-natural chemistries. Curr Opin Chem Biol.

[19] Jeong WY, Kwon M, Choi HE, Kim KS (2021). Recent advances in transdermal drug delivery systems: a review. Biomater Res.

[20] Ansar W, Ghosh S (2013). Monoclonal Antibodies: A Tool in Clinical Research. Indian J Clin Med.

[21] Pasqualini R, Arap W (2004). Hybridoma-free generation of monoclonal antibodies. Proc Natl Acad Sci.

[22] Ryman JT, Meibohm B (2017). Pharmacokinetics of monoclonal antibodies. CPT Pharmacometrics Syst Pharmacol.

[23] Zhang Y, Zhang T, Liu M, Kuang Y, Zu G, Zhang K, Cao Y, Pei R (2018). Aptamer-targeted magnetic resonance imaging contrast agents and their applications. J Nanosci Nanotechnol.

[24] Zhu H, Zhang L, Liu Y, Zhou Y, Wang K, Xie X, Song L, Wang D, Han C, Chen Q (2016). Aptamer-PEG-modified Fe3O4@Mn as a novel T1- and T2- dual-model MRI contrast agent targeting hypoxia-induced cancer stem cells. Sci Rep.

[25] Pusuluri A, Krishnan V, Lensch V, Sarode A, Bunyan E, Vogus DR, Menegatti S, Soh HT, Mitragotri S (2019). Treating tumors at low drug doses using an aptamer-peptide synergistic drug conjugate. Angew Chem Int Ed Engl.

[26] Kim M, Kim DM, Kim KS, Jung W, Kim DE (2018). Applications of cancer cell-specific aptamers in targeted delivery of anticancer therapeutic agents. Molecules.

[27] Hori SI, Herrera A, Rossi JJ, Zhou J (2018). Current advances in aptamers for cancer diagnosis and therapy. Cancers (Basel).

[28] Santulli-Marotto S, Nair SK, Rusconi C, Sullenger B, Gilboa E (2003). Multivalent RNA aptamers that inhibit CTLA-4 and enhance tumor immunity. Cancer Res.

[29] Berezhnoy A, Stewart CA, McNamara JO, Thiel W, Giangrande P, Trinchieri G, Gilboa E (2012). Isolation and optimization of murine IL-10 receptor blocking oligonucleotide aptamers using high-throughput sequencing. Mol Ther.

[30] McNamara JO, Andrechek ER, Wang Y, Viles KD, Rempel RE, Gilboa E, Sullenger BA, Giangrande PH (2006). Cell type-specific delivery of siRNAs with aptamer-siRNA chimeras. Nat Biotechnol.

[31] Chabner BA, Roberts TG (2005). Timeline: chemotherapy and the war on cancer. Nat Rev Cancer.

[32] Yang GH, Lee YB, Kang D, Choi E, Nam Y, Lee KH, You HJ, Kang HJ, An SH, Jeon H (2021). Overcome the barriers of the skin: exosome therapy. Biomater Res.

[33] Hughes B (2010). Antibody–drug conjugates for cancer: poised to deliver. Nat Rev Drug Discov.

[34] Webb S (2011). Pharma interest surges in antibody drug conjugates. Nat Biotechnol.

[35] Hu R, Zhang X, Zhao Z, Zhu G, Chen T, Fu T, Tan W (2014). DNA nanoflowers for multiplexed cellular imaging and traceable targeted drug delivery. Angew Chem Int Ed Engl.

[36] Huang F, You M, Chen T, Zhu G, Liang H, Tan W (2014). Self-assembled hybrid nanoparticles for targeted co-delivery of two drugs into cancer cells. Chem Commun (Camb).

[37] Ducry L, Stump B (2010). Antibody-drug conjugates: linking cytotoxic payloads to monoclonal antibodies. Bioconjug Chem.

[38] Zhu G, Niu G, Chen X (2015). Aptamer-Drug Conjugates. Bioconjug Chem.

[39] Galmarini CM, Mackey JR, Dumontet C (2002). Nucleoside analogues and nucleobases in cancer treatment. Lancet Oncol.

[40] Chen X, Zhou L, Wang J, Jiang G, Cheng H, Pei R (2016). The study of the interaction between doxorubicin and single-stranded DNA. ChemistrySelect.

[41] Richards AD, Rodger A (2007). Synthetic metallomolecules as agents for the control of DNA structure. Chem Soc Rev.

[42] Ebrahimi SB, Samanta D, Cheng HF, Nathan LI, Mirkin CA (2019). Forced intercalation (FIT)-aptamers. J Am Chem Soc.

[43] Zhu G, Chen X (2018). Aptamer-based targeted therapy. Adv Drug Deliv Rev.

[44] Yoon S, Huang KW, Reebye V, Mintz P, Tien YW, Lai HS, Sætrom P, Reccia I, Swiderski P, Armstrong B, Jozwiak A, Spalding D, Jiao L, Habib N, Rossi JJ (2016). Targeted delivery of C/EBPα -saRNA by pancreatic ductal adenocarcinoma-specific RNA aptamers inhibits tumor growth in vivo. Mol Ther.

[45] Yoon S, Huang KW, Reebye V, Spalding D, Przytycka TM, Wang Y, Swiderski P, Li L, Armstrong B, Reccia I, Zacharoulis D, Dimas K, Kusano T, Shively J, Habib N, Rossi JJ (2017). Aptamer-drug conjugates of active metabolites of nucleoside analogs and cytotoxic agents inhibit pancreatic tumor cell growth. Mol Ther Nucleic acids.

[46] Shangguan D, Li Y, Tang Z, Cao ZC, Chen HW, Mallikaratchy P, Sefah K, Yang CJ, Tan W (2006). Aptamers evolved from live cells as effective molecular probes for cancer study. Proc Natl Acad Sci U S A.

[47] Shangguan D, Cao Z, Meng L, Mallikaratchy P, Sefah K, Wang H, Li Y, Tan W (2008). Cell-specific aptamer probes for membrane protein elucidation in cancer cells. J Proteome Res.

[48] Shangguan D, Tang Z, Mallikaratchy P, Xiao Z, Tan W (2007). Optimization and modifications of aptamers selected from live cancer cell lines. Chembiochem.

[49] Lau A, Bérubé G, Ford CH, Gallant M (1995). Novel doxorubicin-monoclonal anti-carcinoembryonic antigen antibody immunoconjugate activity in vitro. Bioorg Med Chem.

[50] Willner D, Trail PA, Hofstead SJ, King HD, Lasch SJ, Braslawsky GR, Greenfield RS, Kaneko T (1993). Firestone RA: (6-Maleimidocaproyl) hydrazone of doxorubicin--a new derivative for the preparation of immunoconjugates of doxorubicin. Bioconjug Chem.

[51] Huang YF, Shangguan D, Liu H, Phillips JA, Zhang X, Chen Y, Tan W (2009). Molecular assembly of an aptamer-drug conjugate for targeted drug delivery to tumor cells. Chembiochem.

[52] Mongelard F, Bouvet P (2010). AS-1411, a guanosine-rich oligonucleotide aptamer targeting nucleolin for the potential treatment of cancer, including acute myeloid leukemia. Curr Opin Mol Ther.

[53] Rosenberg JE, Bambury RM, Van Allen EM, Drabkin HA, Lara PN, Harzstark AL, Wagle N, Figlin RA, Smith GW, Garraway LA (2014). A phase II trial of AS1411 (a novel nucleolin-targeted DNA aptamer) in metastatic renal cell carcinoma. Investig New Drugs.

[54] Bates PJ, Kahlon JB, Thomas SD, Trent JO, Miller DM (1999). Antiproliferative activity of G-rich oligonucleotides correlates with protein binding. J Biol Chem.

[55] Bates PJ, Laber DA, Miller DM, Thomas SD, Trent JO (2009). Discovery and development of the G-rich oligonucleotide AS1411 AS a novel treatment for cancer. Exp Mol Pathol.

[56] Soundararajan S, Wang L, Sridharan V, Chen W, Courtenay-Luck N, Jones D, Spicer EK, Fernandes DJ (2009). Plasma membrane nucleolin is a receptor for the anticancer aptamer AS1411 in MV4-11 leukemia cells. Mol Pharmacol.

[57] Chandola C, Neerathilingam M. Aptamers for targeted delivery: current challenges and future opportunities. India: IntechOpen; 2018.

[58] Trinh TL, Zhu G, Xiao X, Puszyk W, Sefah K, Wu Q, Tan W, Liu C (2015). A synthetic aptamer-drug adduct for targeted liver Cancer therapy. PLoS One.

[59] Li W, Chen H, Yu M, Fang J (2014). Targeted delivery of doxorubicin using a colorectal cancer-specific ssDNA aptamer. Anat Rec (Hoboken, NJ : 2007).

[60] He J, Peng T, Peng Y, Ai L, Deng Z, Wang XQ, Tan W (2020). Molecularly engineering Triptolide with aptamers for high specificity and cytotoxicity for triple-negative breast Cancer. J Am Chem Soc.

[61] Brinker AM, Ma J, Lipsky PE, Raskin I (2007). Medicinal chemistry and pharmacology of genus Tripterygium (Celastraceae). Phytochemistry.

[62] Li F, Lu J, Liu J, Liang C, Wang M, Wang L, Li D, Yao H, Zhang Q, Wen J, Zhang ZK, Li J, Lv Q, He X, Guo B, Guan D, Yu Y, Dang L, Wu X, Li Y, Chen G, Jiang F, Sun S, Zhang BT, Lu A, Zhang G (2017). A water-soluble nucleolin aptamer-paclitaxel conjugate for tumor-specific targeting in ovarian cancer. Nat Commun.

[63] Bagalkot V, Farokhzad OC, Langer R, Jon S (2006). An aptamer-doxorubicin physical conjugate as a novel targeted drug-delivery platform. Angew Chem Int Ed Engl.

[64] Jeong HY, Kim H, Lee M, Hong J, Lee JH, Kim J, Choi MJ, Park YS, Kim SC (2020). Development of HER2-Specific Aptamer-Drug Conjugate for Breast Cancer Therapy. Int J Mol Sci.

[65] Kratschmer C, Levy M (2017). Effect of chemical modifications on aptamer stability in serum. Nucleic Acid Ther.

